# Clinical, Biological, and Radiological Findings and Management of Lower Respiratory Tract Infections in a Tertiary Hospital in Romania

**DOI:** 10.7759/cureus.67685

**Published:** 2024-08-24

**Authors:** Irina Bulata-Pop, Ioana Stirbu, Bianca Simionescu, Alina Grama, Lia Monica Junie

**Affiliations:** 1 Microbiology, “Iuliu Hatieganu" University of Medicine and Pharmacy Cluj-Napoca, Cluj-Napoca, ROU; 2 Neurology, "Iuliu Hatieganu" University of Medicine and Pharmacy Cluj-Napoca, Cluj-Napoca, ROU; 3 Pediatrics, "Iuliu Hatieganu" University of Medicine and Pharmacy Cluj-Napoca, Cluj-Napoca, ROU; 4 2nd Pediatric Discipline, "Iuliu Hatieganu" University of Medicine and Pharmacy Cluj-Napoca, Cluj-Napoca, ROU; 5 Microbiology, "Iuliu Hatieganu" University of Medicine and Pharmacy Cluj-Napoca, Cluj-Napoca, ROU

**Keywords:** antibiotics therapy, respiratory viral infections, bronchitis in children, bronchiolitis, pneumonia, lower respiratory tract infections in children, respiratory tract, infectiuous diseases, pediatrics

## Abstract

Background

Lower respiratory tract infections (LRTIs) remain a significant concern in pediatrics due to their substantial burden among childhood diseases. Romania has recently attained the status of a high-income country. Even though the mortality rate from respiratory diseases has significantly declined from 24.1 per 100,000 individuals in 2000 to 5.3 per 100,000 in 2022, the rate remains notably higher than the European average. Diagnosing LRTI is challenging due to its clinical similarity to noninfectious respiratory illness and frequent false-positive results or incidental findings on microbiologic tests. This often leads to antimicrobial overuse and adverse outcomes. Additionally, antibiotic resistance poses a significant global public health threat.

Patients and method

We conducted a retrospective analysis of pediatric LRTI cases at a tertiary pediatric center in Romania to evaluate diagnostic testing, imaging use, etiology identification, and treatment approaches. Children under 18, admitted to the Emergency Clinical Hospital for Children in Cluj-Napoca during an eight-month peak respiratory season, were included. Data from electronic medical records were analyzed for demographics, symptoms, physical exams, laboratory data, presence of fever, etiology, treatment, and outcomes.

Results

In total, 222 children were included in this study, with a median age of 29 months. Among the participants, 58% were male. The average hospital stay was 11 days. The total number of cases was almost equally split between bronchiolitis and pneumonia, with lobar pneumonia accounting for 12% of the included patients. We found two statistically significant correlations between the presence of fever, intercostal retracting, and the subtype of LRTI. An inflammatory response defined as an elevated leucocyte count and elevated C-reactive protein (CRP) was more likely to appear in pneumonia cases than bronchiolitis. From a therapeutic point of view, the prescription of hydrocortisone was statistically linked to bronchiolitis, but its use did not shorten hospitalization time.

Conclusion

Policy interventions and targeted treatments can reduce LRTI incidence and improve outcomes. Based on our correlations between specific clinical traits and subtypes of LRTIs, the use of assessment scores in children helps predict severe illness and the need for hospitalization. Promoting hygiene, social distancing, and addressing socioeconomic factors are crucial. Larger sample sizes and advanced diagnostics are needed to refine treatment strategies further. Early antibiotic use in children has long-term health implications, including a higher risk of respiratory-caused premature death in adulthood. This emphasizes the need for improved diagnostic processes and specific etiological identification, with metagenomics showing promise in this area.

## Introduction

Lower respiratory tract infections (LRTI) are one of the most challenging pathologies in children, remaining the main cause of morbidity and mortality in the zero- to five-year-old age group. LRTIs are infections caused by viruses, bacteria, or, rarely, fungi that affect the airways below the level of the larynx (including the trachea and the alveolar sacs). From a clinical point of view, LRTI includes tracheitis, bronchitis, pneumonia, and bronchiolitis [1]. According to the World Health Organization (WHO), LRTIs are recognized as the leading infectious cause of death globally and disproportionately affect children [2]. Pneumonia kills more children than any other infectious disease, claiming the lives of over 700,000 children under five every year, or around 2,000 every day. This includes around 190,000 newborns. Almost all these deaths are preventable. WHO provides guidelines [3] for the identification of pneumonia based on simple clinical signs and strategies for treatment management. However, there are concerns that children with non-severe pneumonia are still receiving antibiotics unnecessarily [4].

Reporting the annual incidence of pneumonia is challenging because many cases are treated on an outpatient basis. In Romania, the rate remains significantly above the European average. Data provided by UNICEF about pneumonia (last update: November 2023) shows a 25% mortality rate in children under five years of age. Although the trend is decreasing [5], these numbers are similar to the level present in low-income countries like Angola, Chad, and Nigeria, almost twice as high as our neighbors Bulgaria and Hungary.

Local statistics are crucial for characterizing LRTI clinical findings, improving diagnostic tests, and assessing treatment outcomes. Differentiating between diseases without diagnostic tests is challenging due to overlapping symptoms, making it difficult to distinguish noninfectious causes and bacterial from viral LRTI. Given the limitations of performing complete diagnostic workups, empirical approaches are necessary.

LRTI involves a complex interplay between pathogens, the lung microbiome, and the host response, which is not fully captured by current diagnostics. This complexity contributes to inappropriate antimicrobial use [6], exemplified by the overtreatment of acute bronchitis, which leads to multidrug resistance in pathogens like Streptococcus pneumoniae [7].

The WHO criteria for diagnosing pneumonia [3] in children (cough or difficulty breathing plus tachypnea) are effective in identifying pneumonia from upper respiratory tract infections but are not as sensitive when it comes to differentiating LRTI from non-infectious wheeze [4]. A prospective study conducted in Brazil on children under the age of 59 months examined the efficiency of these criteria. The results indicate that WHO criteria have high sensitivity to detect pneumonia, especially among patients aged <24 months. The specificity, however, was very poor for wheezing children. Adding fever to this set of criteria improved specificity substantially with little loss in sensitivity, avoiding the risk of undertreating children with pneumonia [4].

Identifying pneumonia is both straightforward and complex. Most doctors feel confident making the diagnosis [8] but agree that defining pneumonia involves a complex discussion about pathological, radiological, microbiological, and clinical aspects.

LRTIs result from pathogens entering the respiratory system, causing inflammation in the airways and alveoli, and potentially extending into the interstitial space.

Without pathological examination, pneumonia is typically diagnosed based on chest radiographs showing acute infection changes [9]. While chest ultrasound and MRI are promising, their clinical effectiveness and impact on outcomes need further research.

Standard diagnostic tests like bacterial culture have limited sensitivity and a narrow identification spectrum [10], compounded by challenges in obtaining accurate sputum or bronchoalveolar lavage samples. Rapid multiplex tests on upper respiratory samples can detect pathogens but struggle to determine if they are causative or merely colonizing. Current biomarkers cannot reliably distinguish between bacterial, mixed bacterial-viral, and viral infections. New biomarkers based on host transcriptional profiles show promise but require validation [11].

Children are often incidental carriers of pathogenic microbes [12], leading to false positives and antibiotic overuse. This stands in contrast to adults, who have lower rates of incidental carrier status in both clinical and metagenomic studies.

For patients under five years old, the most common cause of illness is viral, with RSV being the leading cause of bronchiolitis, followed by adenovirus and influenza virus [13]. These infections show a seasonal pattern, occurring mainly in winter and spring or during the first rainy season (March to May) in some regions [14]. Other viral agents identified include enterovirus, parainfluenza, and human metapneumovirus (hMPV), though they are mentioned in fewer studies due to limited testing availability. Streptococcus pneumoniae is the most frequent bacterial cause of LRTI.

In children over five years old, the viral causes are more evenly distributed among RSV, rhinovirus, influenza, hMPV, and adenovirus. The range of bacteria varies by country, depending on available vaccinations [15]. In developing countries, the most common causes of pneumonia are Streptococcus pneumoniae, tuberculosis, and, in severe cases, Haemophilus influenzae and Gram-negative bacteria like Klebsiella pneumoniae and Pseudomonas aeruginosa.

In most cases, patients with LRTI need supportive care that includes maintenance of adequate hydration, relief of nasal congestion/obstruction, and monitoring for disease progression [3].

For immune-competent infants and children with nonsevere bronchiolitis who can be treated as outpatients, routine pharmacologic interventions are not recommended. These have no proven benefits and may increase costs and risk of adverse effects. Current literature does not support using bronchodilators, glucocorticoids, or leukotriene inhibitors. Antibiotics should only be prescribed if a bacterial infection is present [16]. This approach is endorsed by the American Academy of Pediatrics, the National Institute for Care Excellence, and other professional organizations [3,17,18].

Given the fact that LRTI is a complex pathology with a high incidence in the pediatric population, our study’s main goal is to offer an overview of the clinical aspects of LRTI in the geographical region, assess the diagnostic resources, and identify the etiology, along with an analysis of the treatment strategies used, focusing on possible glucocorticoid and antibiotic overuse.

The most recent national studies include the reporting of a case of metapneumovirus infection in 2007 [19], an article on the viral etiologies of respiratory infections in children published in 2013 [20], and a comparative study in two centers on the use of antibiotic treatment in childhood pneumonia published in 2018 [21].

## Materials and methods

Study setting

We performed a retrospective study on patients admitted to the Second Pediatrics Clinic, Emergency Clinical Hospital for Children, Cluj-Napoca. The period chosen was at the peak of the respiratory infectious season, from October 2017 to May 2018, before the SARS-CoV-2 pandemic.

Study design and patient selection

The study aimed to offer oversight on the clinical aspects of LRTI, the diagnostic and etiological tools used, and the management of the patients. Patients between 0 and 18 years of age who presented with respiratory symptoms and met the WHO criteria for LRTI [5] were included.

Lower respiratory tract diseases include [4]: (i) pneumonia - a pulmonary infiltrate on a chest X-ray (CXR) plus fever and/or respiratory complaints. (ii) Acute bronchitis - cough and sputum after an upper respiratory tract infection (URTI) of less than three weeks, without wheezing, and no previous diagnosis of asthma. (iii) Acute bronchiolitis - cough, breathlessness, tachypnoea, wheezing, crepitations, and pulmonary hyperinflation on CXR, following URTI in children <2 years. (iv) Tracheitis: the inflammation of the trachea, usually secondary to a nose or throat infection, fever, toxic appearance, stridor, tachypnea, and respiratory distress. The cough is frequent and not painful.

Non-infectious diseases include: (i) wheezing - the first or second episode of wheezing without other characteristic signs or symptoms. (ii) Recurrent wheezing - children with wheezing and a history of at least two similar episodes.

Chronic patients with a history of asthma or allergic bronchitis and non-infectious diseases were excluded.

We retrospectively applied the Paediatric Respiratory Severity Score (PRESS), a tool used in triaging patients for the assessment of respiratory status. PRESS was developed by Thokngaen et al. and validated on a group of 120 patients aged 3 months to 14 years old. The aim of the study was to assess the sensitivity and specificity of hospitalization. The secondary outcomes are sensitivity, specificity of intensive care unit (ICU) admission, the duration of oxygen therapy and nebulized bronchodilator, using the following criteria: respiratory rate, wheezing, accessory muscle use, peripheral oxygen saturation (SpO_2_), and feeding difficulties. The PRESS score is classified into three groups: mild (score of 0 or 1), moderate (score of 2 or 3), and severe (score of 4 or 5) [22]. Higher scores are linked to hospitalization and oxygen supplementation requirements. The retrospective use of this score intends to verify how many of the cases would have been red-flagged and consequently admitted in a triage scenario, thus allowing for lower admittance ratios in the future.

Data collection

Data from the digital medical records regarding demographics (gender, age, weight), underlying medical conditions, symptoms at admission, laboratory results, microbiological testing, radiological findings, treatment, and duration of stay were collected.

For variables like fever, respiratory rate, and SpO_2_, we used the study of Thokngaen et al. as a guideline [22]. We considered fever as the body temperature above 38 °C; for SpO_2_, we used 92% as the cut-off. For respiratory rate, we defined a high respiratory rate as more significant than 60 breaths per minute for infants under 12 months. For children aged 12-36 months, a normal respiratory rate was considered to be less than 40 breaths per minute. In the 36-156 month age group, a respiratory rate over 30 breaths per minute was deemed pathological, and for those older than 156 months, a threshold of 20 breaths per minute was applied.

During hospitalization, in order to identify the etiology of the LRTI, several methods were commonly used: microbiological culture from sputum, where it was possible to obtain it; bronchoalveolar lavage samples; or blood cultures. Serological tests detecting antibodies or antigens in the blood were used for *Mycoplasma pneumoniae*, *Chlamydia pneumoniae*, Epstein-Barr virus, and Cytomegalovirus. For viral etiologies like influenza and rotavirus, adenovirus, rapid tests were performed. At the time of the study, no test for respiratory syncytial virus (RSV) was available.

Chest X-rays were used in order to help identify characteristic patterns of infection, such as lobar pneumonia, which can suggest specific etiologies.

Statistical analyses

Statistical analyses were performed using Microsoft Excel 2406 and SPSS Statistics V.23 (Microsoft® Corp., Redmond, WA). The chi-square and Mann-Whitney U tests were used to compare different groups. For assessing relationships between variables, we used Spearman’s rank correlation and ANOVA tests, verified by Tukey's HSD (honestly significant difference) test. Statistical significance was set at a p-value of <0.05.

Ethics

This research was conducted according to the principles of the Declaration of Helsinki. All patients admitted to the hospital consented to participate in medical research, including retrospective studies, at the time of their admittance. All patient data obtained were anonymized, and ethics approval was not needed according to Romanian law.

## Results

Population characteristics

Patients' demographic characteristics are summarized in Table 1.

**Table 1:** Demographic data SD: standard deviation. This table includes the median age, gender distribution, and the average hospital stay in days. The standard deviation for gender is not applicable.

	All patients (n=222)
Age, months (mean ± SD)	29 (34.2)
Female (number, %)	94 (42.3)
Male (number, %)	128 (57.6)
Average hospital stay, days (mean ± SD)	11 (5.65)

Symptoms and clinical findings

The frequency of symptoms and clinical findings for all LRTI subtypes are described in Table 2.

**Table 2:** Frequency of symptoms and clinical findings for all LRTI subtypes Disease: This column lists the types of lower respiratory tract infections (LRTIs) diagnosed in the study's pediatric population. Sample size (n): The number of children diagnosed with each type of LRTI in the study. Age (mean months): The average age of the children in months for each disease category. Length of stay (mean days): The average number of days the children were hospitalized. Prior antibiotic treatment (%): The percentage of children who received antibiotic treatment before being hospitalized. Symptoms: Lists common symptoms observed in the children, with percentages indicating how frequently each symptom occurred within each disease group. RR: respiratory rate. Clinical findings: Details observed clinical signs related to respiratory infections, with percentages showing the frequency in each disease group. SpO_2_ < 92%: Percentage of patients with oxygen saturation levels below 92%, indicating potential hypoxemia or oxygen deficiency in the blood.

	Bronchiolitis (n=100, 45%)	Pneumonia (n=90, 40.5%)	Lobar pneumonia (n=27, 12%)	Bronchitis (n=1, 0.5%)	Bronchopneumonia (n=4, 1.8%)	P-value
Age (mean, months)	13	41	48	48	39	0.000193
Length of stay (mean, days)	10	12	13	4	15	0.161320
Prior antibiotic treatment	4(4%)	19 (21.1%)	4 (14.8%)	0	0	<0.0001
Symptoms						
Wet cough	70 (70%)	50 (55.5%)	17 (62.9%)	0	3 (75%)	<0.0001
Dry cough	30 (30%)	40 (44.4%)	10 (37%)	1 (100%)	1 (25%)	<0.0001
Fever	47 (47%)	67 (74.4%)	25 (92.5%)	1 (100%)	4 (100%)	<0.0001
Rhinitis	96 (96%)	89 (98.8%)	20 (74%)	1 (100%)	4 (100%)	<0.0001
Wheezing	25(25%)	8 (8.8%)	0	0	1 (25%)	<0.0001
Dyspnea	21(21%)	20 (22.2%)	4 (14.8%)	0	1 (25%)	<0.0001
High RR	32 (32%)	36 (40%)	4 (14.8%)	0	1 (25%)	<0.0001
Nasal flaring	5 (5%)	0	0	0	0	0.000499
Intercostal retraction	59 (59%)	28 (31.1%)	3 (11.1%)	0	1 (25%)	<0.0001
Digestive symptoms	3 (3%)	3 (3.3%)	3 (11.1%)	0	0	0.213680
Clinical findings						
Hoarse bronchial sounds	30 (30%)	39 (43.3%)	7 (25.9%)	0	4 (100%)	<0.0001
Low-pitched rales (coarse crackles)	12 (12%)	43 (47.7%)	12 (44.4%)	0	3 (75%)	<0.0001
High-pitched rales (fine crackles)	68 (68%)	40 (44.4%)	2 (7.4%)	0	2 (50%)	<0.0001
Ronchi	12 (12%)	8 (8.8%)	0	0	0	<0.0001
Wheezing	77 (77%)	37 (41.1%)	3 (11.1%)	0	1 (25%)	<0.0001
SpO2< 92%	21 (21%)	24 (26.7%)	1 (3.7%)	0	1 (25%)	<0.0001
Dehydration	6 (6%)	7 (7.7%)	3 (11.1%)	0	0	0.008248

The correlation coefficient between age and the duration of hospitalization indicates a weak positive correlation, suggesting that there is a slight tendency for older patients to have longer hospital stays, although it is not statistically significant. There was no difference in the duration of stay regarding the LRTI subtype.

The correlation between a particular subtype and the presence of various symptoms was analyzed. We performed an ANOVA test that returned an F-statistic of 6.56 and a p-value of 0.0000532, significantly below the conventional threshold of 0.05, indicating that there were statistically significant differences in recorded temperature values among different LRTI subtypes. We also observed that lobar pneumonia and bronchopneumonia showed high frequencies of fever, with 81% and 75% of cases, respectively. 64% of patients with pneumonia had fever, while in patients with bronchiolitis, the temperature was normal in 62%.

**Figure 1:**
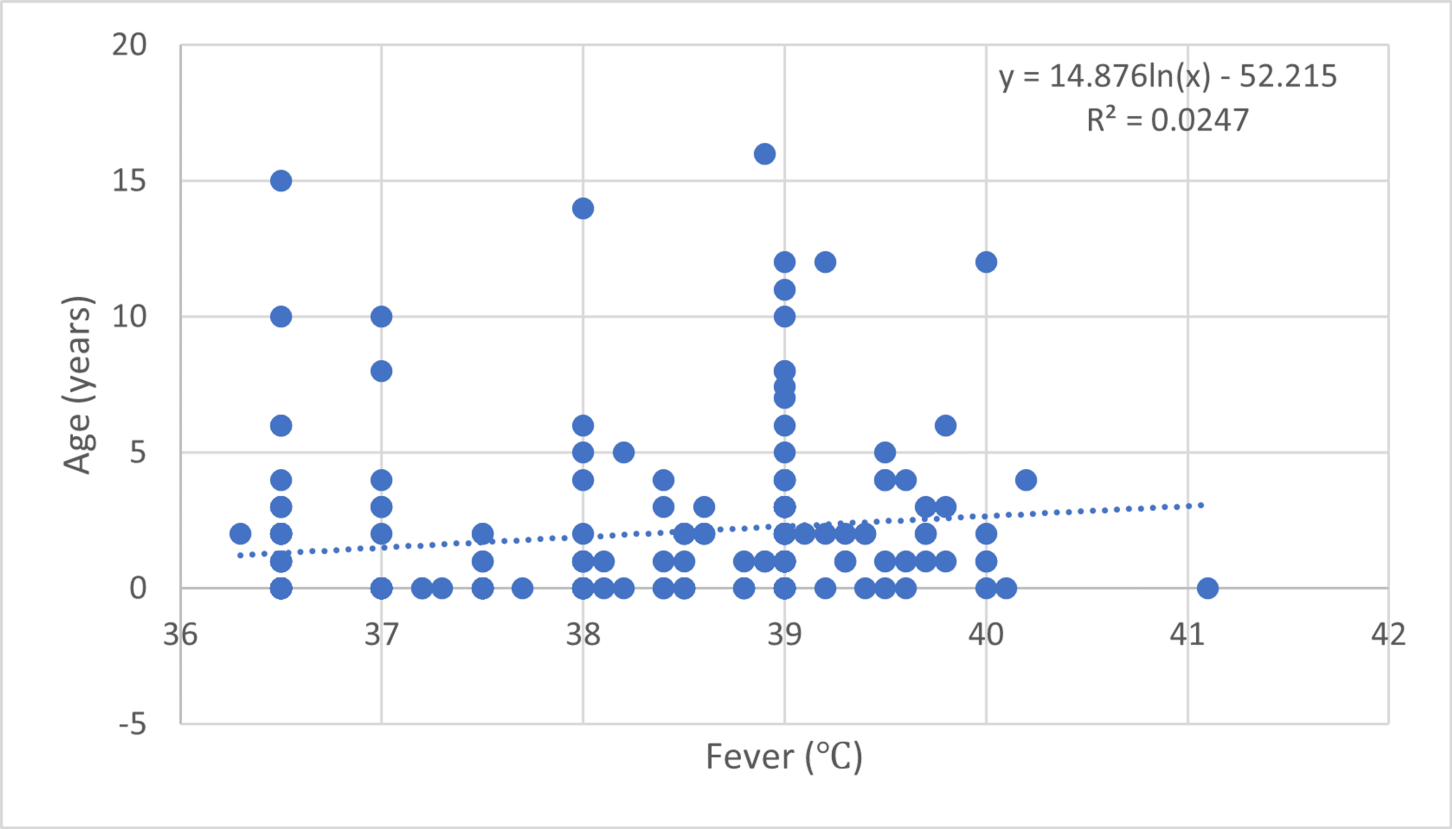
Regression for fever distribution according to age X-axis (fever °C): Represents the body temperature (fever) of patients, ranging from approximately 36 °C to 41 °C. Y-axis (age years): Represents the age of the patients in years, ranging from 0 to 20 years. The equation for the trendline is given as: y = 14.876 × ln⁡(x) − 52.215*y* = 14.876\times\ln(x) − 52.215y = 14.876 × ln(x) − 52.215. R-squared (R²) value: 0.0247.

However, performing the Mann-Whitney U test to identify the possible difference in fever presence for the group of patients that received antibiotic treatment prior to hospitalization compared to those without antibiotics, we obtained a p-value of 0.003. Higher levels of fever were also correlated to the presence of rhinitis (Mann-Whitney U test, p-value = 0.002) and age, as shown in Figure 1.

Talking about signs and symptoms, we did not identify any correlation between wheezing, the type of cough, dyspnea, high respiratory rate, oxygen level or nasal flaring, and a particular LRTI subtype.

**Figure 2:**
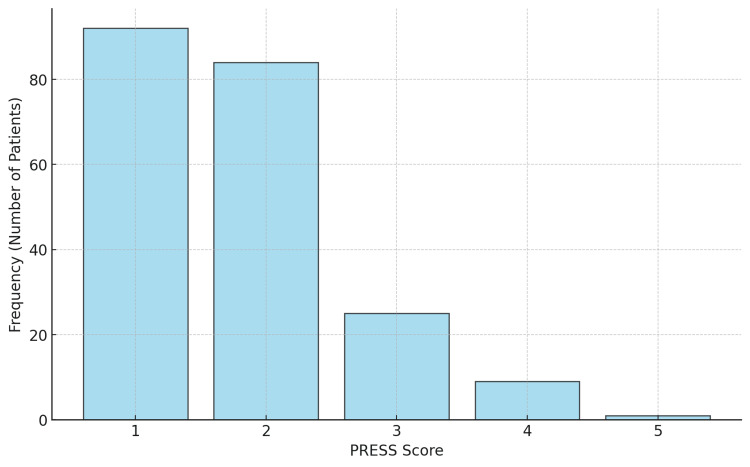
PRESS score distribution The histogram represents the distribution of PRESS scores among patients, reflecting the frequency of each score. X-axis (PRESS score): Displays the PRESS scores, which range from 1 to 5. Y-axis (frequency - number of patients): Indicates the number of patients corresponding to each PRESS score. The PRESS score is classified into three groups: mild (score of 0 or 1); moderate (score of 2 or 3); severe (score of 4 or 5).

However, the presence of intercostal retractions in bronchiolitis was statistically significant (p-value = 0.000271). The Chi-square statistic (19.02) suggests that the observed differences between expected and actual frequencies are large enough to conclude a significant association. We retrospectively applied the PRESS score to the results shown in Figure 2.

**Figure 3:**
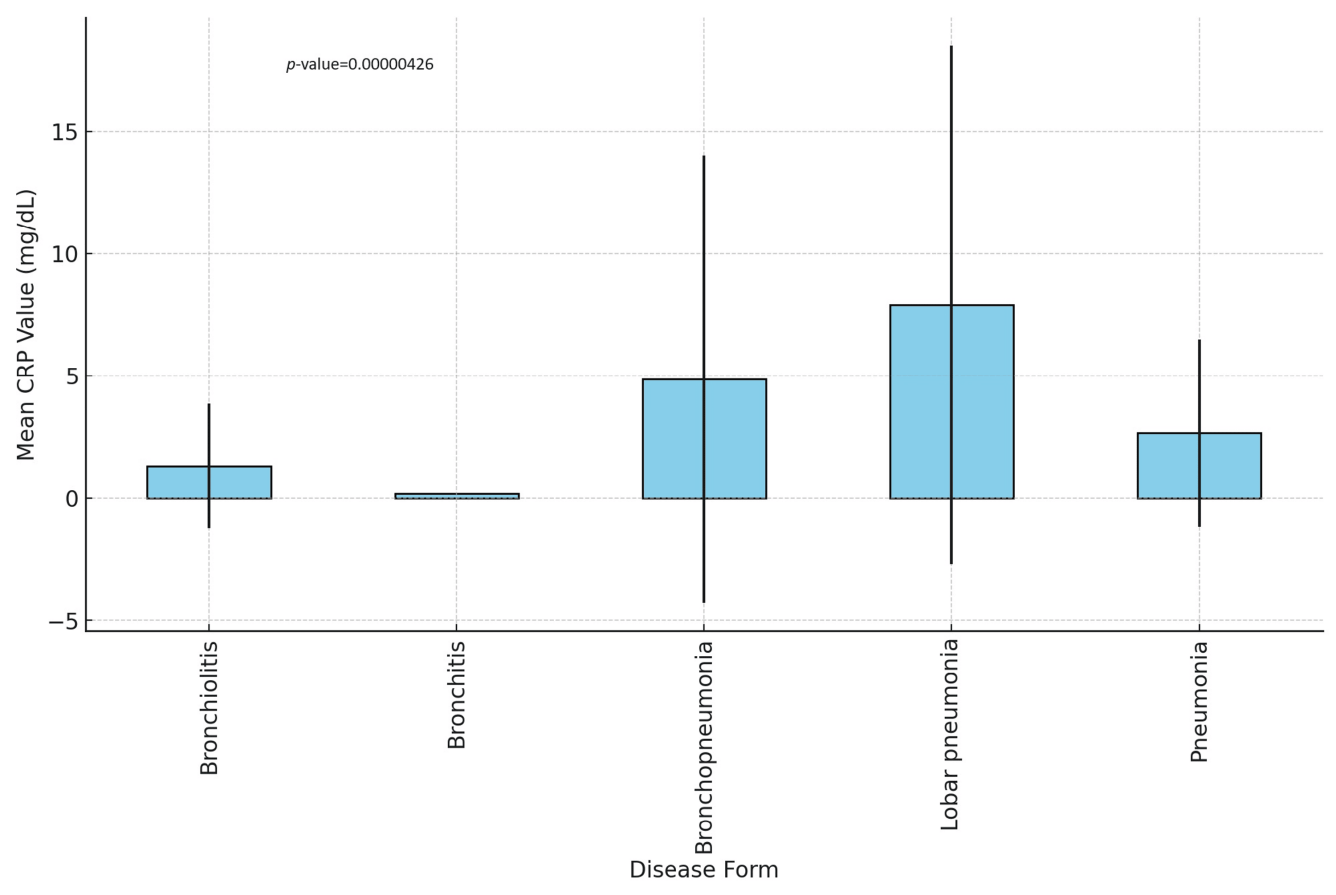
C-reactive protein variation among LRTI subtypes CRP: C-reactive protein (mg/dL). X-axis (disease form): lists the different disease forms being analyzed. Y-axis (mean CRP value in mg/L): shows the average CRP value for each disease form. Error bars: Represent the standard deviation, providing insight into the spread of CRP values around the mean. *P*-value is the result of the one-way ANOVA test used to compare the means of PCR levels across different disease types.

We performed a Pearson correlation test, but the p-value of 0.3724 indicates that this correlation is not statistically significant. We assessed the presence of an inflammatory response in relation to the clinical outcome. We used the ANOVA test on C-reactive protein (CRP) and leukocyte levels. The first correlation yielded a p-value of 0.00426, significantly below 0.05, linking levels of CRP above 1 mg/dl to pneumonia, lobar pneumonia, and bronchopneumonia, as shown in Figure 3. The F-statistic (7.68) suggests that the variation in CRP levels between the groups is greater than would be expected by chance.

**Figure 4:**
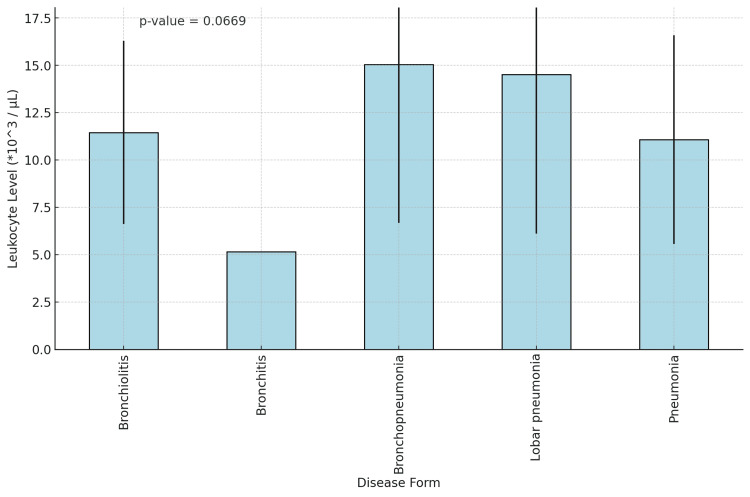
Leukocyte level correlated to the type of disease Leukocyte normal range (*10^3^ /µL): Total leukocytes (children >2 years) = 5-10. Total leukocytes (children aged 2 years or below) = 6.2-17. Error bars represent the standard deviation. The p-value from the ANOVA test is included at the top of the plot.

Regarding the leukocyte level correlated to the type of disease, the p-value obtained was 0.044, showing that in lobar pneumonia, there are higher and more variable leukocyte levels compared to other diseases, while in pneumonia, there is great variability among patients, as seen in Figure 4.

In order to identify risk factors, we performed regression analysis for different variables. The only significant results were a positive coefficient of 3.4851 for higher levels of leucocytes on admission in lobar pneumonia and a positive coefficient (5.6330) for CRP in LRTI subtypes that have a more frequent bacterial etiology.

**Figure 5:**
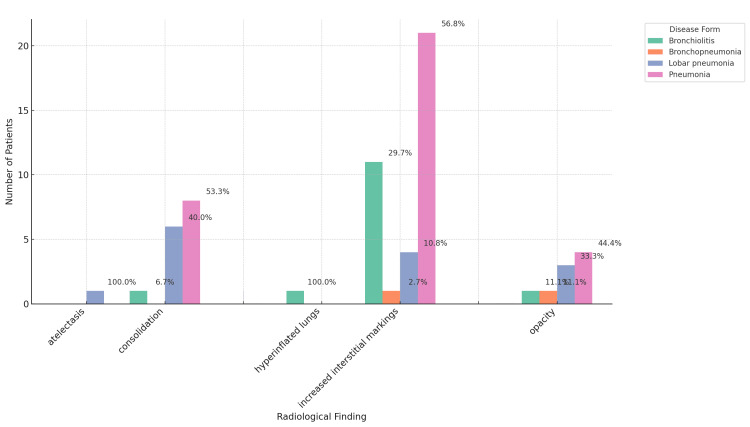
Chest radiography results Bar plot displaying the frequency of radiological findings, separated by disease form, in patients where this information was available or a radiography was performed. The percentage in the columns represents the number of patients with a particular radiological finding within a specific disease form.

When it comes to radiological findings, 66 of the patients had a recorded chest radiography result (29%). The major changes are illustrated in Figure 5.

**Figure 6:**
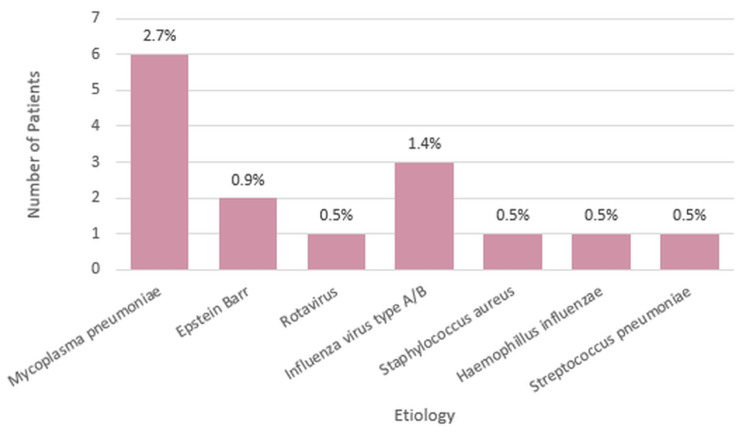
Identified etiology The distribution of identified etiologies among the patient population, identified by microbiological culture from sputum or bronchoalveolar lavage samples, serological tests for *Mycoplasma pneumoniae* and Epstein-Barr virus. For viral etiology, like influenza and rotavirus, rapid tests were performed. Y-axis (number of patients): Represents the count of patients associated with each etiology. X-axis (etiology): lists the different etiologies identified. Percentage labels: Each bar is annotated with the percentage that this etiology represents out of the total patient population. This percentage gives context to the relative frequency of each etiology within the dataset.

Out of the 222 patients, only 15 of them (7%) have an identified etiology through serology or via culture from a bronchoalveolar aspirate. Figure 6 gives an overview of the etiology.

The treatment options are illustrated in Table 3.

**Table 3:** Treatment strategy This table reflects the frequency (number of patients) of each treatment across the different disease forms, along with the associated p-values to indicate the statistical significance of these differences.

Treatment	Lobar pneumonia	Pneumonia	Bronchopneumonia	Bronchiolitis	Bronchitis	p-value
2nd generation cephalosporin	4	14	2	13	0	0.35637
3rd generation cephalosporin	21	60	3	20	1	<0.0001
Ampicillin/amoxicillin	1	6	0	3	0	0.776062
Macrolide	7	13	2	1	0	0.000404
Gentamicin/amikacin	3	8	1	3	0	0.207702
Hydrocortisone	19	61	2	90	0	0.000549
Salbutamol	10	54	4	83	0	<0.0001
Fluticasone	20	73	3	96	1	0.005838

We observed a positive correlation between the use of hydrocortisone and the disease type, in particular bronchiolitis (p-value = 0.000461). Nonetheless, the correlation coefficient of −0.073 suggests that the use of hydrocortisone has a minimal impact on hospitalization duration.

## Discussion

This study is one of the few studies focusing on LRTI in Romania [19-21] and certainly the first in the last five years. Through our research, we managed to offer a wider perspective on the clinical spectrum and the importance of the etiology guiding the treatment strategy.

The study highlights significant correlations between LRTI subtypes and certain clinical features, such as fever and inflammatory markers. Literature supports similar findings: the presence of fever, abnormal pulmonary auscultation or chest retractions, or being assessed as “unwell” increased the likelihood of a pneumonia diagnosis by at least fivefold in a Danish study [23] following patients in the same period of time as our study. Additionally, even a slightly elevated CRP (≥11 mg/L) was positively associated with a pneumonia diagnosis.

A study published in 2023 [24] reported a high sensitivity (98%) of tachypnea in diagnosing pneumonia cases, but with a specificity of only 6%. This only highlights the difficulty in differentiating LRTI and the possible benefit of using prediction scores, such as PRESS, that include multiple clinical markers. This could impact future treatment strategies and help mitigate antibiotic overuse.

Non-pharmaceutical interventions (NPi) and targeted treatments can reduce LRTI incidence and improve outcomes [25]. Promoting hand hygiene, mask-wearing, and social distancing during outbreaks, alongside implementing policies for sick leave and remote work/school options, can significantly reduce the number of LRTIs. Addressing risk factors through health education programs for caregivers with the help of socioeconomic interventions to alleviate poverty, provide nutritional support, and ensure access to clean water and sanitation would lessen the burden on secondary medical care and improve pediatric LRTI mortality rates.

Further research with larger sample sizes and targeted advanced diagnostic methods, such as multiplex PCR tools in severely ill children, is needed to refine the need for hospitalization and improve judicial antibiotic use.

The study has several limitations, including a small sample size of 222 children, which may limit the statistical power of the analysis. Conducted in a single tertiary hospital in Romania, the results may not apply to other settings or populations. The retrospective design relies on existing medical records, which may be incomplete or biased. The study also lacked comprehensive diagnostic testing, with only 7% of patients having an identified etiology, potentially leading to incomplete diagnoses. Additionally, the focus on hospitalized children may not capture the full spectrum of LRTI severity, and the absence of long-term follow-up limits the assessment of long-term outcomes.

Although LTRI is generally seen as a common event in pediatrics, in recent years, the use of antibiotics early in life has been thought to have an impact on surviving LRTI episodes during adulthood. One study observing premature death in Great Britain [26] aimed to evaluate the association between early childhood LRTI and the risk and burden of premature adult mortality from respiratory disease. Data showed that adults with a history of early LRTI had an almost two times higher risk of premature death due to respiratory disease. In pediatrics, the lack of rapid methods for an exact etiology and difficulty obtaining sputum generally translate into more pressure on clinicians to recommend antibiotics.

Therefore, identifying specific etiologies remains challenging and worth pursuing, considering they might impact patients' long-term health alongside the risks of antibiotic overuse. The ongoing need to optimize the diagnostic process for LRTIs has drawn the attention of specialists in metagenomics [27]. The study by Heitz et al. compared metagenomic next-generation sequencing (mNGS) and culture. mNGS identified additional pathogens potentially implicated in pneumonia without etiologic diagnosis by culture. These findings are similar to the ones in the Mick et al. study [6]: "Lower airway metagenomics has the potential to detect host and microbial signatures of LRTI. However, its scalability and applicability for enhancing diagnosis and treatment in a pediatric population remain uncertain."

## Conclusions

This study revealed concordant results to the literature, underlining the high incidence of cases in a relatively short period of time, with a small number of identified etiologies, but with antibiotic treatment for approximately half of the patients.

A comprehensive strategy involving NPIs, socioeconomic improvements, healthcare access, and targeted health education can significantly reduce LRTI incidence and improve outcomes in children, especially during epidemics. Early antibiotic use in children has implications for long-term health. Therefore, optimizing diagnostic processes and identifying specific etiologies remain critical in trying to reduce unnecessary antibiotic and glucocorticoid use.
